# Micro- and Nanoplastics Exposure Across the Lifespan: One Health Implications for Aging and Longevity

**DOI:** 10.3390/jox16020052

**Published:** 2026-03-19

**Authors:** Chantalle Moulton, Anna Baroni, Ennio Tasciotti

**Affiliations:** 1 Human Longevity Program, IRCCS San Raffaele Roma, 00166 Rome, Italy; chantalle.moulton@sanraffaele.it (C.M.); anna.baroni@sanraffaele.it (A.B.); 2 Department of Human Sciences and Promotion of the Quality of Life, San Raffaele Roma University, 00166 Rome, Italy

**Keywords:** microplastics, nanoplastics, One Health, aging, exposome, environmental pollution, inflammaging, rehabilitation

## Abstract

Micro- and nanoplastics (MNPs) are pervasive environmental contaminants with growing relevance for human health across the lifespan. Older adults may be especially vulnerable to their effects due to cumulative lifetime exposure, age-related physiological changes, and a higher burden of chronic disease. Adopting a One Health perspective, this review synthesizes current evidence on the sources, exposure pathways, and biological effects of MNPs, integrating findings from environmental, animal, and human studies with a specific focus on aging populations. Experimental studies consistently show that MNP exposure triggers oxidative stress, inflammation, mitochondrial dysfunction, and cellular senescence, mechanisms central to biological aging. These processes are linked to dysfunction of the cardiovascular, nervous, gastrointestinal, and immune systems, suggesting that MNPs may contribute to the development or progression of age-related diseases. Within the One Health framework, MNPs also act as carriers of chemical additives and environmental pollutants, potentially amplifying health risks through combined and cumulative exposures along food chains and ecosystems. Despite increasing mechanistic evidence, direct epidemiological data in older adults remain limited. This review highlights key knowledge gaps and emphasizes the need for integrative, longitudinal research to clarify the role of MNPs in aging and to inform public health and environmental policy.

## 1. Introduction

Plastic is a consistent feature of modern life, but the practical advantage based on its long-lasting nature has also led to the emerging issue of global “plasticization” of ecosystems, where the progressive fragmentation of plastic waste has generated micro- and nanoplastics (MNPs) across air, water, soil, and food webs [1].

The reason why MNPs are challenging xenobiotic stressors is that, in addition to their ubiquity, they are also extremely diverse, in terms of size, shape, polymer chemistry, and weathering state [2] (Wright et al., 2024). Moreover, MNPs can interact with polymer additives, adsorb and transport heavy metals, persistent organic pollutants, microbes, and other environmental contaminants, acting as mobile vectors that facilitate their entry into organisms and tissues where these compounds may subsequently desorb and enhance toxicity [3,4,5,6,7]. This real-world complexity confounds exposure reconstruction, dosimetry, and causal inference, yet the pervasiveness of contamination implies that, under plausible chronic exposure scenarios, even small individual-level risks could translate into substantial population-level burdens, especially in aging societies [8,9,10].

MNPs are increasingly recognized as contaminants of global relevance within a One Health framework. One Health is an integrated, unifying approach that recognizes that the health of humans, animals, and ecosystems are interconnected and must be addressed collaboratively across disciplines to effectively prevent and manage health threats [11,12]. It promotes coordinated actions among fields such as medicine, veterinary science, environmental science, and public health to address complex issues including zoonotic diseases, antimicrobial resistance, and environmental degradation [11,13].This framework is useful to understand the scale and persistence of plastic production and the capacity of plastic debris to fragment, disperse, and enter food webs. Current production and consumption patterns ensure that plastic waste will remain in the environment for decades, progressively generating globally distributed reservoirs of MNPs [14,15]. Their integration into trophic networks links ecosystem integrity, animal health, and human exposure, particularly through dietary routes [16]. The detection of MNPs in food, drinking water, and human biological samples raises concern that chronic, low-dose exposure may have subtle but systemic effects that develop over long timescales [17,18]. Although analytical advances now allow movement beyond simple detection toward quantification of dose and persistence, limited standardization and persistent contamination issues continue to constrain population-level risk assessment [19].

Aging provides a biologically relevant context for evaluating MNP exposure, as environmental insults accumulate across the life course and may interact with age-related declines in physiological reserve. As resilience decreases with age, comparable exposures can exert greater functional consequences, amplifying vulnerability at the population level [20,21]. Older adults therefore represent cohorts with substantial cumulative exposure via inhalation and ingestion, even when daily doses are low [22]. The fate of internalized MNPs likely depends on particle size, shape, polymer chemistry, and surface properties, yet environmentally realistic toxicokinetic data remain limited [23]. Concurrently, aging is characterized by immunosenescence, chronic low-grade inflammation, mitochondrial dysfunction, impaired repair capacity, and altered barrier integrity. Furthermore, accelerated aging refers to the premature accumulation of molecular hallmarks of aging, such as genomic instability, epigenetic alterations, mitochondrial dysfunction, and chronic inflammatory signaling, occurring at a faster rate than expected for chronological age [24]. Mechanistic studies of MNP exposure repeatedly report oxidative stress, inflammatory signalling, immune dysregulation, mitochondrial damage, and cellular senescence, closely aligning with pathways central to biological aging and age-related disease susceptibility [25,26].

The relevance of later life is further strengthened by the high prevalence of multimorbidity and polypharmacy, reflecting reduced physiological reserves across multiple organ systems [27,28]. Because rehabilitation in the elderly occurs in the context of pre-existing frailty, multimorbidity, and limited recovery capacity, MNP research must extend beyond general populations to address how these exposures may interfere with recovery trajectories in already compromised individuals. In this context, additional environmental stressors that promote inflammation, endothelial dysfunction, or oxidative stress may accelerate disease progression, even if they are not primary causal agents. Emerging evidence suggests that MNPs can modulate pathways involved in vascular, endothelial, and neurobiological dysfunction, supporting their consideration as modifiers of age-related disease trajectories [26,29]. Exposure across the lifespan is also affected by a heterogeneous set of causes, shaped by socio-economic conditions, residential and occupational histories, and indoor environments, creating inequalities in cumulative exposure and vulnerability. This mirrors patterns observed for other environmental stressors, such as heat and air pollution, where older adults experience disproportionate impacts driven by multimorbidity and altered physiology [20,21,30,31]. Importantly, focusing on aging does not imply deterministic causality, but reflects a biologically informed hypothesis that MNP exposure may influence the pace of biological aging through convergence on pathways of inflammation, mitochondrial dysfunction, and functional decline [32].

A One Health perspective is essential to integrate environmental, animal, and human evidence, recognizing MNPs as shared exposures linking ecosystem health with animal well-being and human longevity. Although originally emphasized in infectious disease contexts, One Health is increasingly applied to chronic disease prevention and healthy aging, reflecting the interdependence of safe environments with human longevity [33,34,35]. MNP exposure spans ecosystems and species, with wildlife and domestic animals encountering similar ingestion and inhalation routes that shape human exposure patterns [27,36,37]. Comparative studies indicate that pollution and habitat degradation can accelerate aging-related phenotypes via oxidative stress, inflammation, and immune dysfunction, supporting the relevance of conserved mechanisms [38,39]. Evidence of MNP-associated gastrointestinal, metabolic, and immune disturbances in non-human models may therefore inform human aging while underscoring ecosystem-level harm [40].

Within this framework, MNPs also challenge conventional toxicology. A geroscience-informed One Health lens conceptualizes pollutants as potential accelerators of aging processes and emphasizes reduced compensatory capacity in older organisms exposed to cumulative, multisystem stressors [34,41]. Interpretation is further complicated by environmental weathering, biofilm formation, and co-exposure to other pollutants, all of which can modify particle reactivity and obscure classical hazard attribution [42,43,44].

The objective of this review is therefore to evaluate MNPs as persistent, low-dose xenobiotics within an aging population using a One Health approach, by reviewing the most recent findings in the literature, and give a review of what is currently known about these contaminants. Here, we synthesize evidence on MNP sources, environmental fate, and exposure routes as well as mechanistic data linking MNP exposure to processes relevant to aging [14,29,45,46,47]. By combining environmental, animal, and human evidence, this review highlights shared mechanisms and identifies key gaps relevant to causality and risk assessment [48,49,50,51]. Given the threat that these MNPs present to environmental, animal, and, most importantly, human health, this research is essential to better understand and map the situation to remedy the identified negative effects.

## 2. Defining Micro- and Nanoplastics

Due to the fact that MNPs are still governed by operational, method-dependent definitions, it is necessary to state how these terms are used in this review. In practice, boundaries are constrained by analytical detectability, and differences in reporting metrics (particle number vs. mass), polymer identification, and surface characterization can limit cross-study comparability and exposure–response inference [52,53,54].

This definitional problem is not ‘simply a semantic issue’. It shapes exposure assumptions, determining which particles are represented in experimental models, and influences how mechanistic results are translated into risk, particularly in aging research, where barrier integrity, clearance capacity, and sensitivity to chronic low-dose stressors may change across the life course [55,56,57].

### 2.1. Terminology and Size Classifications Definitions

Microplastics (MPs) have most frequently been described as solid plastic particles between 5 mm and 1 µm in size [58]. This size-based convention is now widely adopted across environmental monitoring and toxicology, although refinements increasingly emphasize that MPs are synthetic or heavily modified polymers that are persistent, insoluble, and particulate [1]. By contrast, nanoplastics (NPs) lack a single agreed definition. A commonly used working definition includes particles in the range of 1 nm to 1 μm in size that display colloidal properties like Brownian motion, high specific surface area, and high affinity to natural colloids [59,60,61].

Importantly with respect to aging biology, this operational differentiation has functional significance because with smaller size in the sub-micrometer range, there is an increased probability of translocation, cell internalization, and subcellular interactions, and thus a shift in fundamental questions from irritation and barrier function effects linked with MPs, towards immune recognition, mitochondria disruption, and relevance/retention effects with NPs [62,63,64].

MNPs are also frequently categorized by origin as primary or secondary. Primary particles are intentionally manufactured at micro- or nanoscale and deliberately added to personal care products, industrial abrasives, specialized applications, and pre-production pellets/powders [65,66,67,68]. Secondary particles result from the successive erosion of larger plastic debris, comprising packaging, textiles, tires, paints, and marine debris, facilitated by mechanical abrasion, UV radiation, thermal cycling, and biological activity [69,70,71].

For health-relevant risk interpretation, secondary particles are particularly important because they typically dominate environmental burdens and exhibit highly heterogeneous morphologies (irregular fragments, films, fibres) and complex “exposure histories” that include prolonged weathering, additive leaching, sorption of co-pollutants, and biofilm colonization [72,73,74].

Due to the fact that secondary MNPs accumulate complex exposure histories (weathering, additive loss, pollutant sorption, and biofilm growth), their surface properties and reactivity can differ markedly from pristine materials; accordingly, environmentally aged particles are typically considered more exposure-relevant test materials than pristine, uniform spherical beads frequently used in experimental studies [75].

### 2.2. Physicochemical Properties Related to Aging Biology

#### 2.2.1. Size, Shape, Polymer Type and Surface Chemistry

Particle size, shape, polymer type, and surface chemistry control the translocation of MNPs through the environment and interactions with biological barriers after oral or pulmonary administration [64,76]. Smaller particles, below the micrometer scale, exhibit higher surface area and colloidal stability and tend to more successfully cross biological barriers and reach secondary organs after administration compared with the same mass concentrations above the micrometers scale [77].

Shape further influences aerodynamic and cellular contact parameters: fibres and irregular shapes can exert more mechanical stresses at the expense of different biological barriers and tend to induce more severe site inflammation compared to spherical MNP with the same exposed surface area under similar administration conditions [1,78].

The type of polymer can also be important due to differing densities, crystallinity, and additive composition between common types (e.g., PE, PP, PS, PVC, PET) [14,79]. Surface chemistry is dynamic: weathering processes (surface oxidation and surface fractures) can change hydrophobicity, surface charge properties, surface topography, and sorption properties, potentially impacting interactions with heavy metals, persistent organic pollutants, and biological surfaces [43,80].

Even so-called “eco-friendly” or biodegradable plastics are not inherently safe at the micro- and nanoscale. Polylactic acid (PLA) MNPs cross biological barriers in mice, accumulate in the male reproductive tract, disrupt the blood–testis barrier, damage sperm mitochondria, and impair spermatogenesis, reducing sperm count and motility in vivo [81]. In zebrafish and C. elegans, pristine and especially UV- or photo-aged PLA MNPs induce oxidative stress, mitochondrial dysfunction, apoptosis, and transgenerational reproductive toxicity at environmentally relevant µg–mg/L concentrations [82,83,84,85]. In vitro, PLA and other biodegradable MNPs can alter gut microbiota after simulated digestion and are internalized by human cell lines, where PLA and polybutylene succinate (PBS) particles trigger cytotoxicity and oxidative stress [86,87]. Reviews further report that PLA, polycaprolactone (PCL) and polybutylene adipate terephthalate (PBAT) can cause developmental, reproductive, immune and endocrine disruption across multiple models, sometimes with toxicity comparable to conventional plastics [88,89,90]. Together these findings indicate that reduced carbon footprint does not necessarily imply lower toxicological risk.

Given the pivotal role of both oxidative stress and inflammation in particle-mediated responses as well as biological aging, these factors are not simply environmental details, as they might impact the extent and type of immune system dysregulation and age-related physiological impairment [91].

#### 2.2.2. Aging, Weathering, and Bio-Corona Formation

In natural conditions, multi-factor aging processes of MNPs are driven by UV irradiation, temperature cycling, mechanical wear, redox reactions, and biological activity [63,78]. Across studies, aging commonly results in surface fragmentation, the introduction of oxygen-containing functional groups (e.g., hydroxyl and carbonyl), and changes in roughness and crystallinity, which can promote further fragmentation and increase the fraction of potentially penetrating particles [43,72,92]. Aging can also increase contaminant adsorption, for example, smaller and more oxidized particles may exhibit greater capacity to adsorb metals under certain environmental conditions, increasing the likelihood of mixture exposures [93,94,95].

Concurrently, MNPs rapidly acquire an eco-corona or bio-corona, which are layers of natural organic matter, proteins, lipids, and microorganisms (including biofilms) that redefine particle identity from the perspective of cells and tissues [96,97]. After uptake in organisms, the way this bio-corona is progressively remodelled as micro- and nanoplastics transit from the intestine into the bloodstream and onward to organs such as the liver or brain, and how these shifting molecular coats modulate recognition and handling of the particles by aged immune cells, remain critical open questions for future investigation [98].

In aquatic systems, adsorption of organic materials may influence the degree of aggregation, stability, and fate [99], while the natural aging process allows colonization by bacterial populations whose profiles are determined by environmental factors [100,101]. Additionally, in biological fluids, coronas can alter the recognition mechanism between immune and endothelial cells, the uptake mechanism, and the modification of the redox signalling pathways may be influenced, which is related to inflammaging and increased susceptibility to endothelial damage with age [102,103,104]. Combined, these various factors reinforce the need for incorporating environmentally relevant particle aging and corona development into experimental studies and risk assessments, particularly those that seek to understand how low doses may interact with declining physiological resilience factors in older populations [80,96].

## 3. Environmental Sources and Distribution Across the One Health Continuum

**Figure 1:**
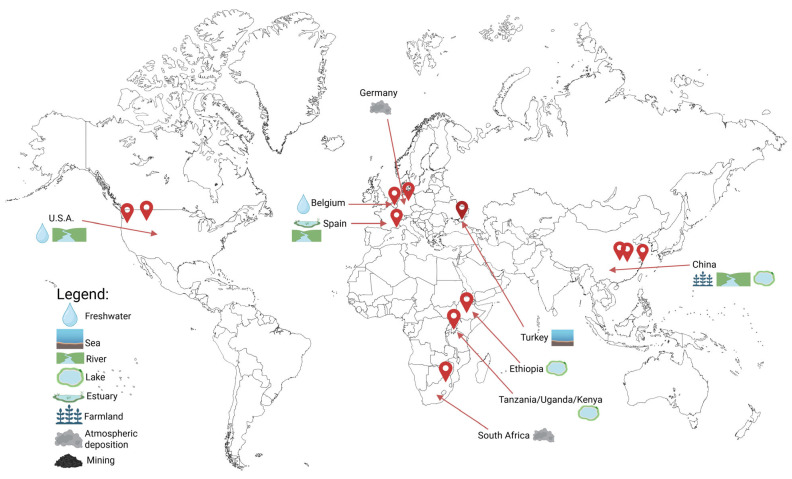
Identified micro- and nanoplastics (MNPs) across the globe in different environmental compartments, such as rivers, lakes, and soils [109,110,111,112,113,114,115,116,117,118,119,120,121]. The map summarizes geographic locations where MNPs have been detected in environmental matrices including freshwater systems (rivers and lakes), marine environments, estuaries, agricultural soils, mining areas, and areas affected by atmospheric deposition. Symbols represent the environmental compartment in which MNPs were reported. These findings illustrate the extensive environmental dissemination of MNPs and their ability to circulate across interconnected terrestrial, aquatic, and atmospheric systems. Within a One Health perspective, this widespread distribution underscores the potential for MNPs to move through ecosystems and food chains, ultimately contributing to human exposure.

MNPs are now pervasive across air, water, soil, and biological systems, creating continuous exposure for humans, livestock, wildlife, and ecosystems within a One Health context (Table 1). Their widespread presence results from sustained global plastic production, environmental fragmentation, and inefficient waste management, followed by long-distance atmospheric and hydrological transport across interconnected environmental compartments (Figure 1) [105,106,107,108].

### 3.1. Environmental Compartments and Transport

MNPs are consistently detected in the atmosphere, seawater, rivers, lakes, estuaries, sediments, and aquatic organisms across all continents, confirming global distribution and persistence [105,107,108]. Rivers, stormwater, and runoff act as major hydrological arteries that transport MNPs from inland and urban environments toward coastal and marine ecosystems, where they accumulate in sediments and biota [106,122]. Several continental-scale syntheses indicate that concentrations in inland waters and stormwater often exceed those reported in downstream estuaries, highlighting strong terrestrial to aquatic gradients and continuous inputs from land-based sources [106,122].

Terrestrial systems function both as long-term reservoirs and as active contributors to onward dispersion. Inputs arise from sewage sludge application, manure, tire wear particles, degraded plastic mulches, and atmospheric deposition, with particles persisting in soils, altering soil structure, and interacting with organisms in soil [122,123,124].

Airborne transport is now recognized as a central component of MNP dispersal, and a major source of xenobiotic exposure via inhalation in humans. MNPs have been detected in urban, suburban, rural, and remote regions, including mountainous and remote environments, confirming long-range atmospheric movement and deposition to land and water systems [105,106,125,126]. Indoor environments are repeatedly highlighted as significant exposure environments for humans because textile fibres and household plastics contribute substantially to indoor air and dust, which is particularly important for vulnerable populations spending prolonged time indoors [123,125,126].

Collectively, converging evidence indicates that MNPs form a pervasive environmental reservoir that links air, soil, freshwater, and marine compartments into a single interconnected exposure continuum [105,106,107,108,125].

### 3.2. Entry into Food Webs and One Health Links

Overall, MNPs enter aquatic food webs through the bioaccumulation documented from plankton to fish, seabirds, and marine mammals. Evidence indicates trophic transfer and retention within biological tissues, supporting concerns regarding long-term accumulation and biological impacts across species [107,123,124,127]. Parallel findings in terrestrial species demonstrate ingestion in livestock and wildlife, with species-specific vulnerability and physiological consequences [36,128,129].

Specifically, within aquatic ecosystems, fish and seafood occupy a pivotal position in the transfer of MNPs from the environment to higher trophic levels, including humans. MNPs have been widely documented in commercially relevant finfish, bivalves, crustaceans, and processed seafood products across diverse geographic regions [127,130,131,132,133]. Particle burdens vary substantially with feeding strategy and trophic position. Filter feeders such as mussels and oysters, as well as shrimp and small fish consumed whole, typically exhibit the highest levels of ingestion because gastrointestinal contents are retained in the edible fraction [130,131,132,133]. In contrast, for most larger finfish where viscera are removed prior to consumption, MNP levels in muscle tissue are generally lower, although contamination can still arise from post-harvest processing and packaging [131,132,134].

Risk evaluations by EFSA and subsequent reviews suggest that, under conservative assumptions, MNPs in seafood currently contribute a relatively minor proportion to overall exposure to plastic-associated additives and contaminants [133,135,136]. However, these assessments are limited by sparse data on particles smaller than 150 μm and on NPs, which may exhibit higher bioavailability. Moreover, MNPs present in seafood can act as vectors for co-occurring contaminants, including plastic additives, metals, hydrophobic organic chemicals, and microorganisms, raising concern for combined toxicological effects along aquatic food webs [37,127,130,131,132,137]. From a One Health perspective, contamination of marine food webs therefore represents a shared challenge linking environmental pollution, fish health, food safety, and human exposure.

Human exposure occurs primarily through ingestion and inhalation. Drinking water, seafood, salt, fruits, vegetables, and processed foods have been shown to contain MNPs, with estimated annual intakes in the tens of thousands of particles per person, although significant methodological uncertainty remains [123,124,138]. Importantly, detection of MNPs in human faeces, placenta, blood, lung and brain tissue, and other biological matrices confirms internal exposure and indicates the potential for bioaccumulation across the lifespan [107,124,139,140,141,142].

**Figure 2:**
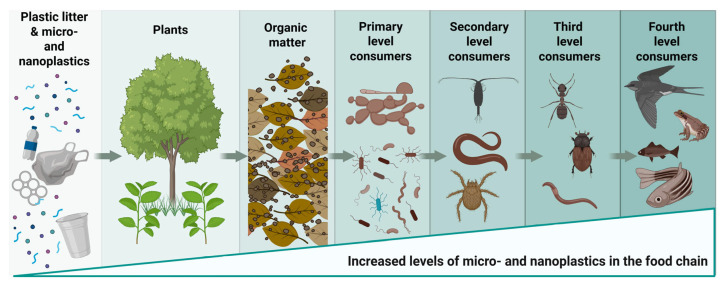
Bioaccumulation of plastic in the food chain due to plastic litter, microplastic and nanoplastic pollution. Environmental plastic debris progressively fragments into micro- and nanoplastics that enter terrestrial and aquatic ecosystems and become incorporated into soils and organic matter. These particles can be taken up by plants or ingested by primary consumers such as soil and aquatic invertebrates. Through trophic transfer, micro- and nanoplastics may move to higher trophic levels, including insects, fish, birds, and other predators. This process illustrates how plastic pollution can propagate through food chains and contribute to indirect exposure in higher organisms, including humans.

Within a One Health perspective, terrestrial domestic animals and companion animals are increasingly viewed as environmental sentinels because they share indoor air exposure, interact with contaminated soils and food chains, and may reflect exposure burdens relevant to human health (Figure 2). Their role in manure to soil to crop pathways is particularly important for agricultural exposure cycles and food security [124,134,141].

## 4. Internalization, Distribution, and Persistence

MNPs can enter the human body through ingestion and inhalation, and a fraction can cross epithelial barriers to reach systemic circulation. Although most exposures likely result in limited absorption, converging human, animal, and in vitro evidence indicates that a proportion of particles, particularly smaller MNPs, can be internalized, distributed to organs, and persist within different tissues for sustained periods. The extent to which these processes contribute to meaningful lifetime body burdens and interact with aging biology remains insufficiently characterized.

### 4.1. Absorption and Barrier Translocation

Ingestion and inhalation currently represent the most established exposure pathways in humans [141,143]. Pharmacokinetic analyses indicate that gastrointestinal absorption is generally low, yet not negligible. Multiple mechanisms permit barrier passage, including transcytosis in enterocytes, uptake by M-cells, persorption through epithelial discontinuities, and phagocytosis by immune cells, with efficiency strongly dependent on particle size, surface charge, and chemistry [141,144,145]. Advanced intestinal co-culture models support the capacity of NPs to cross via passive diffusion and endocytic routes, reinforcing biological plausibility for systemic entry [146].

The respiratory tract represents an additional relevant entry point, with inhalation being the main exposure route to these xenobiotics. Only particles below approximately 5 µm efficiently reach the lower lung, although the presence of larger particles in lung tissue suggests possible vascular entrapment after systemic redistribution rather than direct deposition [141]. Human studies reporting MNPs in lung tissue, sputum, blood, placenta, and brain confirm that barrier translocation occurs in real-world contexts [147,148,149,150]. Collectively, the available evidence supports the conclusion that while the majority of ingested or inhaled MNPs may be cleared through mucociliary or gastrointestinal mechanisms, a consistent fraction of particles can penetrate biological barriers and enter systemic circulation, particularly when they fall within the micro-to-nano size range [141,147,151].

### 4.2. Biodistribution, Persistence, and Accumulation in Aging

**Figure 3:**
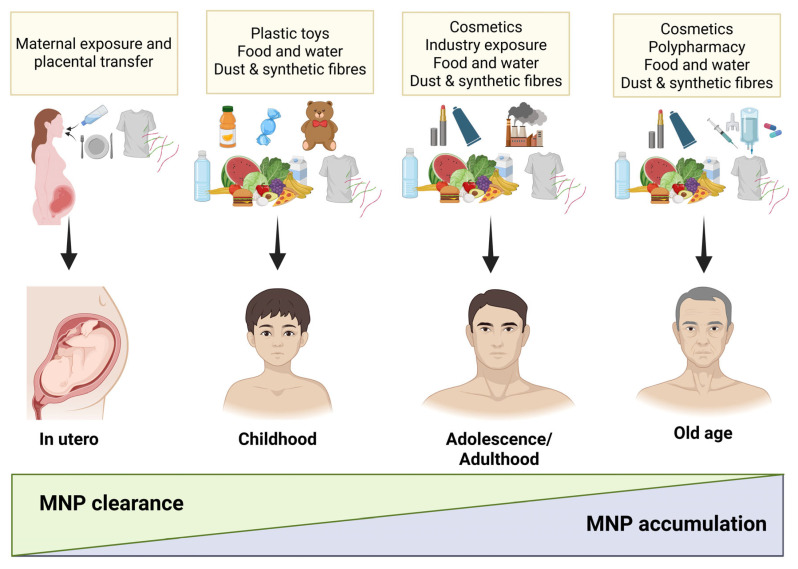
Sources of micro- and nanoplastic (MNP) exposure across the lifespan, including food, synthetic fibres, occupational exposure, and polypharmacy, which may contribute to progressive bioaccumulation as exposure increases and physiological clearance declines over time. Exposure may begin in utero through maternal exposure and possible placental transfer, although evidence documenting direct foetal biological effects of MNPs remains limited [155]. During childhood, exposure may arise from plastic-containing products such as toys, food packaging, drinking water, and indoor dust or fibres. In adolescence and adulthood, additional sources include cosmetics and occupational environments. Across the lifespan, cumulative exposure combined with reduced clearance may promote gradual accumulation of micro- and nanoplastics in the body.

Experimental work in rodents demonstrates that absorbed particles do not remain confined to the entry site but distribute to multiple organs including the liver, kidney, gut, spleen, brain, and reproductive tissues, with smaller particles exhibiting broader distribution and higher tissue retention (Figure 3) [144,152,153]. Physiologically based toxicokinetic models in mice indicate that gastrointestinal absorption and faecal elimination dominate whole-body kinetics, while organ accumulation reflects size-dependent differences in binding and clearance, including reduced urinary elimination for smaller particles [23]. Model estimates indicate that nanoplastics and microplastics (1 to 10 μm) accumulate progressively in human tissues across the lifespan, reaching substantially higher levels in adults than in children. Specifically, cumulative intake is estimated at 8.32 × 10^3^ particles per capita (90% CI, 7.08 × 10^2^ to 1.91 × 10^6^) or 6.4 ng per capita (90% CI, 0.1 to 2.31 × 10^3^) by 18 years of age, whereas accumulation increases to 5.01 × 10^4^ particles per capita (90% CI, 5.25 × 10^3^ to 9.33 × 10^6^) or 40.7 ng per capita (90% CI, 0.8 to 9.85 × 10^3^) by 70 years of age, indicating a substantial age-dependent increase in retained particles in body tissues [154].

Human-directed toxicokinetic assessments propose that most ingested particles are excreted, but a systemically absorbed fraction can accumulate in hepatosplenic organs and potentially the brain, with clearance likely involving macrophage-mediated processes and reticuloendothelial sequestration [141,147,151]. Importantly, existing evidence suggests the possibility of incomplete clearance and long residence times. At the cellular scale, MNPs can be internalized via endocytosis and persist over cell divisions, providing a mechanistic basis for cellular bioaccumulation and potentially chronic intracellular exposure [148,149].

Current knowledge strongly indicates size-dependent fate characteristics. NPs show the greatest capacity for barrier crossing and widespread tissue access, including convincing evidence of central nervous system entry in animal models [144,151,153]. Smaller MNPs demonstrate systemic translocation with accumulation in major metabolic and immune organs and are often associated with more tissue-level alterations [23,153]. Larger MPs are more likely confined to the gastrointestinal tract or become physically trapped within vascular structures, resulting in far more limited systemic distribution [141,143].

The relevance of these processes for aging relies on two intertwined considerations. First, MNPs engage biological pathways linked to the hallmarks of aging [147,156,157]. Second, aging itself alters barrier integrity, immune clearance mechanisms, and physiological resilience. Age-associated increases in gut and lung permeability, reduced macrophage efficiency, and altered inflammatory tone could theoretically enhance absorption, systemic persistence, and downstream tissue vulnerability [144,147,158]. However, these implications remain largely inferential, and more research is required. There is a paucity of direct, age-stratified toxicokinetic data, and lifetime accumulation dynamics remain unclear.

## 5. Cellular and Molecular Mechanisms Relevant to Aging

**Figure 4:**
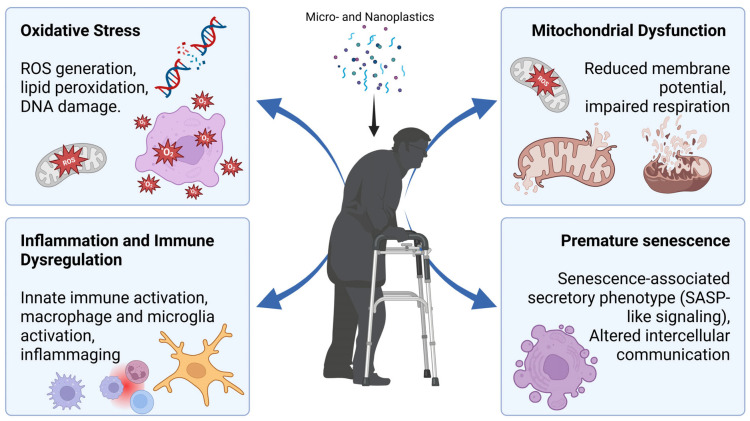
Effects of micro- and nanoplastics (MNPs) on molecular mechanisms relevant to aging. Due to the effects of MNPs at a molecular and cellular level, these particles may contribute to biological aging. MNP exposure may induce oxidative stress, characterized by increased production of reactive oxygen species (ROS), lipid peroxidation, and potential DNA damage. MNPs may also lead to mitochondrial dysfunction, including reduced membrane potential and impaired cellular respiration, which may compromise cellular energy metabolism. In parallel, exposure may promote inflammatory responses and immune dysregulation, including activation of innate immune pathways and macrophage or microglial activation, contributing to chronic low-grade inflammation. Persistent cellular stress may ultimately lead to premature cellular senescence, characterized by the senescence-associated secretory phenotype (SASP) and altered intercellular communication, processes closely linked to the progression of aging-related dysfunction.

MNPs engage several biological pathways that overlap with recognized hallmarks of aging, particularly oxidative stress, chronic inflammation, mitochondrial dysfunction, impaired inter-organelle signalling, and cellular senescence (Figure 4) [156,159,160,161]. While the effects of MNP exposure induce these same pathways, clinical and causative evidence remains scarce, and more evidence is needed to explicitly confirm whether MNP exposure may lead to accelerated biological aging. To date, most mechanistic insights derive from mammalian cell systems and animal models, with limited longitudinal human data, yet the convergence between particle toxicity and aging biology is increasingly documented (Table 2) [156,159,162]. Altogether, however, these preliminary findings warrant the need to investigate how the effects of MNP exposure may affect the hallmarks of aging and accelerated biological aging in vivo.

### 5.1. Oxidative Stress and Redox Imbalance

Across a wide range of models, exposure to MNPs has been shown to induce oxidative stress [163,164]. Studies demonstrate increased production of reactive oxygen species (ROS), depletion of antioxidant systems including glutathione, superoxide dismutase, and catalase, and accumulation of lipid peroxidation, protein oxidation, and DNA damage [156,159,161,162]. NPs are particularly potent, as they can enter mitochondria, impair electron transport chain function, cause membrane depolarization, and promote sustained oxidative stress [159,160,165]. Particle size, surface chemistry, and environmental aging modify these effects, with smaller, positively charged, and UV-aged particles presenting higher redox reactivity [153,161,162].

Aging organisms already exist in a state of weakened antioxidant capacity and heightened oxidative load; therefore this interaction provides strong mechanistic plausibility that chronic low-level exposure could intensify age-related redox imbalance, although robust long-term dose–response data in humans remain unavailable [46,156,159,165].

### 5.2. Inflammation and Immune Dysregulation

MNPs consistently activate innate immune signalling. Experimental work shows activation of NFκB, MAPK, and NLRP3 inflammasome pathways, with associated increases in cytokines such as TNFα, IL1β, IL6, and IL8 across gut, lung, liver, and immune cell systems [156,159,166,167,168,169,170,171,172,173]. Macrophages and dendritic cells internalize particles and show ROS-dependent activation with phenotype shifts [170,172,174,175,176].

This chronic, low-grade immune activation parallels the biology of inflammaging, suggesting that persistent tissue retention of particles could theoretically reinforce inflammatory tone and contribute to immune dysregulation over time. To date, however, direct evidence linking these immune effects to human aging trajectories remains limited and largely inferential [26,156,165,177,178].

### 5.3. Mitochondrial Dysfunction and Metabolic Impairment

Multiple studies indicate that NPs, and in some contexts, small MPs, reach mitochondria where they disrupt structure and function. Reported effects include cristae damage, mitochondrial membrane depolarization, ATP reduction, respiratory chain impairment, calcium imbalance, and excessive mitophagy [147,159,160,165,167,179]. Functional consequences include apoptosis, metabolic disturbance, insulin resistance in muscle systems, and neurodegeneration-like phenotypes in neuronal models [88,159,165,167,179].

Since mitochondrial decline is a central contributor to frailty, neurodegeneration, and metabolic disease in aging, these findings support a biologically plausible pathway through which environmental plastic exposure may exacerbate age-associated mitochondrial vulnerability [88,156,159,160,165].

### 5.4. Cellular Senescence and Altered Cell Communication

Experimental and review evidence indicates that MNPs can promote cellular senescence. Reported mechanisms include oxidative DNA injury, mitochondrial dysfunction, impaired autophagy, activation of DNA damage responses, and p53-related signalling, together with secretion of cytokines consistent with senescence-associated secretory phenotype profiles [156,161,167]. Size-dependent and dosage-dependent mechanisms have been proposed, including ferroptosis and p53–Fosl1–Slc7a11 signalling for NPs, whereas MPs may drive metabolic reprogramming and inflammatory signalling involving YAP pathways [167].

Senescent cell accumulation contributes to tissue dysfunction and systemic inflammaging. Consequently, MNP-induced senescence represents a plausible contributor to accelerated biological aging. However, organism-level confirmation, particularly under environmentally relevant exposure scenarios, remains sparse and represents a major gap in the literature [156,161].

**Table 2:** Experimental evidence linking micro- and nanoplastics to aging mechanisms and hallmarks of aging.

Study/Model	Particle Type & Exposure	Aging-Relevant Cell/Tissue Target	Key Cellular & Molecular Mechanisms	Aging-Linked Outcomes	Citations
Testis multi-omics, young vs. old mice	PS-NPs, chronic oral, 3 months in 3- and 17-month-old mice	Testis, Leydig cells	Age-dependent disruption of RNA metabolism in young vs. DNA catabolism, collagen/ECM remodeling in old; downregulation of SR-BI and impaired steroidogenesis; fibrosis; altered SASP-related pathways	Premature testicular aging in young; aggravated age-related testicular degeneration and sperm decline in old	[168]
Colon toxicity, mice + intestinal epithelial cells	PS 100 nm vs. 10 µm, oral and in vitro	Intestinal epithelium	Nanoscale: endocytosis, ROS, iron overload, GSH depletion, GPX4 inhibition, p53–Fosl1-driven ferroptosis and immunogenic cell death. Microscale: mechanical membrane/cytoskeletal damage, YAP activation, metabolic shift from oxidative phosphorylation to glycolysis, inflammation	Chronic epithelial damage, inflammatory milieu, and metabolic reprogramming associated with tissue aging and cancer risk	[167]
PS-NPs in polyps, microglia, mice	PS-NPs, in vitro and in vivo	Microglia, marine polyps, mouse brain	ROS increase, decreased CAT and T-AOC, increased MDA; MAPK pathway activation; microglial activation and apoptosis; neuroinflammation	Anxiety-like behaviour and cognitive impairment in mice, consistent with accelerated brain aging phenotypes	[180]
PET MPs in accelerated-senescence rats	PET 2–6 µm, 10 or 100 mg/kg, 2 months oral	Brain, lens, retina	Cognitive decline without major blood chemistry changes; progression of cataract and AMD-like retinopathy; mechanisms not fully dissected but linked to chronic exposure	Worsened geriatric phenotypes in OXYS rats, suggesting increased rate of aging and age-related disease burden	[181]
PP MPs in mouse colon	PP 8 and 70 µm, 0.1–10 mg/mL, 28 days oral	Colonic epithelium, barrier	Oxidative stress (redox imbalance), tight-junction disruption, reduced mucus and ion transporter expression; TLR4/NF-κB activation; increased pro-apoptotic and pro-inflammatory proteins, elevated apoptosis	Loss of barrier integrity, chronic inflammation and epithelial apoptosis—hallmarks of inflammaging in gut	[182]
Weathered MPs in adult zebrafish	Naturally weathered PE/PP MPs, 0.1–1 mg/L, 21 days	Brain and liver mitochondria	Decreased mitochondrial complexes II & IV in brain; liver mitochondrial respiration and membrane potential loss; increased SOD/CAT (ROS-induced ROS release)	Anxiety-like behaviour and mitochondrial dysfunction, aligning with mitochondrial theory of aging	[183]
PLA micro/nanoplastics in male mice	PLA MPs → NPs, chronic exposure	Testis, sperm mitochondria, BTB	PLA-NPs cross BTB; excessive mitochondrial ROS, structural damage, impaired mitochondrial function in testes and sperm; transcriptomic suppression of spermatogenesis genes	Reduced sperm quality and hormonal disruption; mitochondrial damage driving reproductive aging	[81]
PS MPs/NPs in retinal cells and rat eye	PS MPs vs. NPs, 48 h in RPE cells; intravitreal injection in rats	Retinal pigment epithelium, retina	NPs internalization → high ROS, mitochondrial fission (FIS1, Drp1), mitophagy (LC3B), ↑SOD2; retinal inflammation with ↑TNF-α, IL-1β	Mitochondrial dysfunction and inflammation in RPE, central mechanisms in age-related macular degeneration	[184]
PS-NPs in zebrafish	PS-NPs ~70 nm, chronic in adults	Brain, liver, gonads	Tissue accumulation; oxidative stress and disturbed lipid/energy metabolism; altered neurotransmitters and circadian rhythm	Neurobehavioral impairment and reproductive toxicity, consistent with systemic functional aging	[185]
PS-NPs in PD mouse model	PS-NPs, 2 mg/kg q.o.d., 3 months, A53T α-syn mice	Gut barrier, microbiota, liver, brain	Goblet-cell loss, epithelial apoptosis; dysbiosis (Desulfovibrio↑); >200 fecal metabolites altered; LPS and apoptosis pathways; liver inflammation; exacerbated neuroinflammation and α-syn aggregation	Acceleration of Parkinson-like pathology via gut–liver–brain axis disruption and metabolic dysregulation	[186]
MPs in female mice and offspring	MPs 40 mg/kg/day, gestation + lactation	Oocytes, embryos, offspring germ cells	Elevated ROS in oocytes/embryos; mitochondrial dysfunction, apoptosis; DNA damage and spindle/chromosome defects; altered actin and Juno	Reduced fertility, impaired offspring growth and oocyte quality—evidence of trans-generational reproductive aging	[187]
Acute PS-MPs in young vs. old mice	PS 0.1 and 2 µm, 3 weeks in water	Brain, liver; age-stratified	Age-dependent behavioural changes; altered immune markers in brain and liver (inflammation); in vitro cytotoxicity with perinuclear accumulation	Short-term exposure induces inflammation and behavioural shifts differing by age, suggesting age-specific vulnerability	[188]
Pre-puberty PS-NPs + cordycepin	PS-NPs 80 nm, 3–12 mg/kg/day (PND 21–95)	Testis, BTB, Sertoli cells	Oxidative stress, BTB disruption (junction proteins↓), inflammation, apoptosis; transcriptomic enrichment of metabolism, lysosome, apoptosis, TLR4 signalling; cordycepin mitigates via TLR4 targeting	Long-term reproductive impairment from early-life exposure; persistent barrier damage and oxidative stress compatible with accelerated gonadal aging	[189]
PS-MPs in chicken kidney	PS-MPs 1–100 mg/L, 6 weeks	Renal mitochondria, tubular cells	Altered mitochondrial dynamics (MFN1/2, OPA1, Drp1), structural damage; oxidative stress (SOD, CAT, MDA, GSH, T-AOC changes); NF-κB activation; necroptosis via RIP1/RIP3/MLKL	Inflammation-driven renal degeneration with necroptosis, mapping onto cell-death mechanisms seen in age-related kidney disease	[164]
UV-aged PS-MPs, C. elegans	Virgin vs. aged PS-MPs, 0.1–100 µg/L, 10 d	Dopaminergic, glutamatergic, serotonergic neurons	Greater neurodegeneration with aged MPs; altered glutamate, serotonin, dopamine levels; dysregulated neurotransmission genes (eat-4, dat-1, tph-1)	Impaired locomotion and neurotransmission, paralleling age-related decline and neurodegeneration	[190]
Aged PS-MPs, zebrafish early life	Pristine vs. UV-aged PS, 0.1–100 µg/L	Embryo/larval mitochondria	Aged MPs → higher ROS, DNA damage, ↓mitochondrial membrane potential, cyt c release; caspase-3/9 activation; transcriptional changes in oxidative stress, mitochondrial dysfunction, apoptosis genes	Developmental defects and mitochondrial apoptosis, pointing to early-life programming of aging pathways	[163]
PS-NPs, dopaminergic neurons & mice	PS-NPs 50 nm, 0.5–500 µg/mL in vitro; 250 mg/kg/d, 28 d in mice	Dopaminergic neurons, mitochondria	NP accumulation in mitochondria; complex I interference; ↓membrane potential, ↓ATP, impaired respiration; AMPK/ULK1-driven excessive mitophagy; ROS-independent cytotoxicity; melatonin rescues	PD-like neurodegeneration and motor deficits via maladaptive mitophagy, a central mechanism in brain aging	[179]
PE-MPs in human discs and models	Environmental PE-MPs; human disc samples, mouse & cell models	Nucleus pulposus cells	MPs detected in human intervertebral discs; in models, TLR4/NOX2 activation → ROS overproduction, oxidative stress, nucleus pulposus cell senescence; TLR4/NOX2 inhibition reverses effects	Disc degeneration through cellular senescence, directly linking MPs to a hallmark of aging	[191]
PS MPs and aging gut microbiome	PS MPs, aged mice	Gut microbiota and fecal metabolome	Loss of beneficial taxa, rise in potentially harmful bacteria; increased metabolites linked to stress and altered host metabolism (e.g., alanine, serine, tryptophan, thymine, methionine, benzoic acid)	Exacerbated gut dysbiosis and aging-associated metabolic signatures, implying higher risk of age-related disease	[192]
MPs ± DEHP in mouse liver	MPs, DEHP, MPs + DEHP	Liver	Antioxidant system impairment; hepatic apoptosis and inflammation; transcriptomic/metabolomic disruption of carbohydrate, amino acid, lipid and purine metabolism; PI3K/AKT activation; induction of hepatocarcinogenesis-related genes	Metabolic dysfunction, chronic inflammation and pro-tumorigenic signalling, overlapping strongly with hepatic aging pathways	[193]

↑, increase; ↓, decrease; MP, microplastic; NP, nanoplastic; PS, polystyrene; PET, polyethylene terephthalate; PP, polypropylene; PE, polyethylene; PLA, polylactic acid; ECM, extracellular matrix; SASP, senescence-associated secretory phenotype; SR-BI, scavenger receptor class B type I; ROS, reactive oxygen species; GSH, glutathione; GPX4, glutathione peroxidase 4; CAT, catalase; T-AOC, total antioxidant capacity; MDA, malondialdehyde; MAPK, mitogen-activated protein kinase; BTB, blood–testis barrier; YAP, Yes-associated protein; TLR4, Toll-like receptor 4; NF-κB, nuclear factor kappa B; RPE, retinal pigment epithelium; TNF-α, tumor necrosis factor alpha; IL-1β, interleukin 1 beta; SOD, superoxide dismutase; MFN1/2, mitofusin 1/2; OPA1, optic atrophy 1; Drp1, dynamin-related protein 1; RIP1/3, receptor-interacting protein kinase 1/3; MLKL, mixed lineage kinase domain-like protein; AMPK, AMP-activated protein kinase; ULK1, unc-51 like autophagy activating kinase 1; PD, Parkinson’s disease; BTB, blood–testis barrier.

Overall, mechanistic evidence indicates that MNPs may activate biological pathways central to aging, including oxidative stress, inflammation, mitochondrial injury, and senescence signalling. This convergence supports biological plausibility that lifelong exposure could interact with aging biology and potentially influence healthspan. However, most studies rely on short-term and relatively high-dose exposures. Determining the relevance of chronic, low-dose environmental exposure for aging outcomes will require aging-focused experimental systems, longitudinal human studies, and integration within life-course exposomics frameworks.

## 6. One Health Implications of Micro- and Nanoplastics for Aging Populations

**Figure 5:**
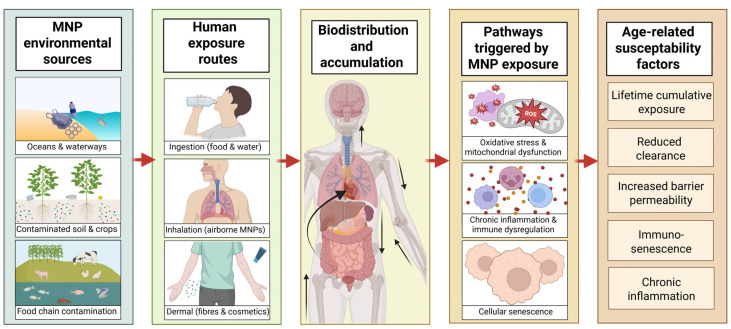
Conceptual framework linking environmental micro- and nanoplastic exposure to aging-related biological effects within a One Health perspective. Micro- and nanoplastics originate from environmental reservoirs such as marine systems, soils, and the food chain, leading to chronic human exposure primarily through ingestion and inhalation. After entering the body, these particles may distribute systemically and accumulate in multiple organs, including the brain, lungs, liver, and gastrointestinal tract. Experimental evidence indicates that MNP exposure can trigger cellular mechanisms associated with aging biology, including oxidative stress, mitochondrial dysfunction, chronic inflammation, immune dysregulation, and cellular senescence. These processes may contribute to functional decline across multiple physiological systems. Age-related factors such as cumulative exposure, reduced detoxification capacity, and immunosenescence may further increase vulnerability in older populations. MNP, Micro- and nanoplastics.

Within a One Health framework, MNPs should be interpreted not merely as ubiquitous environmental contaminants, low-dose stressors that interact with biological aging processes across species. Their relevance for aging populations arises from three converging features: lifelong exposure beginning early in development, physical persistence with limited biodegradation, and mechanistic overlap with conserved pathways that drive age-related functional decline (Figure 5) [55,91]. These characteristics distinguish MNPs from many classical environmental toxicants and justify their consideration within aging and longevity research frameworks to ensure improved healthspan and rehabilitation in older adults.

### 6.1. Ecosystem Health, Animal Sentinels, and Conserved Aging Pathways

MNP contamination affects all major environmental compartments. From a One Health and aging perspective, the critical observation is the common biological responses to MNP exposure across taxa. Laboratory and field studies in aquatic and terrestrial organisms consistently report oxidative stress, immune dysregulation, altered lipid and energy metabolism, mitochondrial dysfunction, and impaired reproduction following chronic exposure [25,194,195]. These pathways overlap substantially with hallmarks of aging, including mitochondrial dysfunction, loss of proteostasis, altered intercellular communication, and stem cell exhaustion [91].

Quantitatively, experimental studies in fish, mollusks, and rodents often report increased reactive oxygen species levels ranging from 20 to 200 percent above controls, accompanied by significant reductions in antioxidant enzyme activity and ATP production, depending on particle size and exposure duration [25,156]. While these exposure levels exceed current environmental estimates, the directionality and consistency of responses support the notion that MNPs activate conserved stress pathways rather than species-specific toxic effects.

From a surveillance standpoint, wildlife and domestic animals exposed to chronic environmental contamination can therefore function as sentinels showing the effects of cumulative biological stress due to MNPs. Observations of reduced reproductive fitness, impaired immune competence, and altered metabolic profiles in animal populations may provide early warnings of processes that, under prolonged exposure and in the context of declining resilience, could influence human aging trajectories.

### 6.2. Aging as a Modifier of Susceptibility in Humans

Aging may fundamentally modify susceptibility to exposure and subsequent internalisation to MNPs. Age-related changes in epithelial barrier integrity, including increased intestinal permeability and reduced mucociliary clearance, may enhance internalization of inhaled or ingested particles [20,196]. Experimental and clinical studies indicate that intestinal permeability can increase in older adults, while immune surveillance and phagocytic capacity decline progressively with age, potentially impairing clearance of persistent particles.

Immunosenescence may further promote persistence of internalized MNPs and sustain low-grade inflammatory signalling [196]. These processes occur against a background of multimorbidity and polypharmacy, although it is still no clear understanding of how this may affect MNP absorption rates. Furthermore, older adults recovering from disease through rehabilitation already face limited physiological reserve, and chronic exposure to MNPs may further hinder successful rehabilitation and healthspan maintenance by sustaining inflammatory and metabolic stress, underscoring the need to explicitly consider these populations in MNP research.

Importantly, aging populations experience cumulative exposure to multiple environmental stressors. Fine particulate matter (PM2.5), for example, has been associated with increased mortality and accelerated biological aging, with long-term exposure above 10 µg/m^3^ linked to measurable reductions in life expectancy [197]. Many of the molecular pathways activated by PM2.5 overlap with those reported for MNPs, including oxidative stress, inflammation, and mitochondrial dysfunction. Within this broader exposomic context, MNPs should be viewed as incremental contributors to physiological burden rather than dominant drivers of pathology.

### 6.3. Public Health, Prevention, and Healthy Aging Frameworks

At the population level, the potential relevance of MNPs for aging emphasizes the importance of preventive environmental health strategies. Although current evidence does not support age-specific exposure thresholds or clinical guidelines, the mechanistic convergence between MNP-induced effects and hallmarks of aging supports their inclusion within healthy aging and longevity research agendas [91].

Primary prevention strategies include reduction of plastic production, more effective recycling, and addressing of environmental pollution. These measures align protection of human health with preservation of ecosystem services and biodiversity. Secondary prevention approaches may involve integration of MNP exposure metrics into exposomic and geroscience cohorts, enabling longitudinal evaluation of associations with biomarkers of biological aging, including epigenetic clocks, chronic inflammation profiles, mitochondrial function, and functional decline [198].

Taken together, these considerations position MNPs as components of a shared ecological and biological context that may modulate aging trajectories across species. A One Health approach therefore provides a scientifically coherent framework for addressing environmental sustainability, animal health, and human healthy aging in an increasingly plastic-dependent world.

## 7. Knowledge Gaps and Future Directions

Substantial uncertainty still limits our ability to link lifelong MNP exposure to aging trajectories and chronic health outcomes, despite increasing biological plausibility. Major gaps exist across exposure science, analytical chemistry, toxicology, and population research.

### 7.1. Limitations in MNP Research and Methodological Challenges in Nanoplastic Detection

Limitations in MNP research arise not only from analytical constraints but also from experimental design. Conflicting results frequently reflect uncontrolled variation in particle type, size, aging, dose, and exposure matrix, which are rarely standardized across studies [199,200,201]. A major bias is the extensive use of monodisperse spherical polystyrene as a model plastic, despite its poor representativeness of heterogeneous environmental polymers, limiting extrapolation to real exposure scenarios [202,203].

Many studies also rely on supra-environmental concentrations, short exposures, and simplified endpoints such as cytotoxicity or oxidative stress, which can overestimate hazard and reduce comparability [201,204,205]. Inconsistent quality control and reporting of particle characterization and experimental conditions further limits reproducibility and risk assessment [8,206]. Together, these issues hinder generalization of findings from idealized nanoplastics to complex environmental mixtures.

Detection and quantification of NPs in environmental and biological samples remains one of the most significant barriers. Spectroscopic approaches such as micro-FTIR and Raman spectroscopy progressively lose discriminatory capability below 1 µm, while thermal analytical platforms including pyrolysis-GC/MS and TED-GC/MS provide polymer mass but do not resolve particle number, morphology, or surface chemistry, especially in complex matrices such as blood or tissues [207,208,209,210,211,212,213]. Reported detections often vary widely across studies, and there is credible concern regarding contamination and false positives, particularly when detection limits may approach or exceed expected environmental exposure levels [143,212,213]. Many studies still lack harmonized quality assurance protocols, including blanks, recovery efficiencies, and standardized reporting units, which limits cross-study comparison and risk assessment frameworks [207,211,212,213]. Ongoing work is exploring enhanced Raman detection, SERS, advanced imaging mass spectrometry, and tracer-labelled plastics to improve traceability across the lifespan [77,207,213]. Parallel development of standardized internal body burden metrics in blood, brain, and other organs is essential to characterize lifetime accumulation and exposure variability [139,140,212,214,215].

### 7.2. Need for Aging-Focused Experimental and Clinical Models

Most toxicological knowledge is derived from experiments using young animals, short exposures, or simplified polystyrene spheres at concentrations that often exceed realistic human levels [25,37,144,204]. Experimental work rarely incorporates aging physiology, despite clear mechanistic overlaps between MNP toxicity and processes such as inflammaging, mitochondrial decline, and cellular senescence [144,156,204]. There is a strong need for aged animal models, disease-susceptible organisms, senescent cell systems, and aged human organoid platforms. Lifespan-oriented study designs spanning prenatal development to late life, combined exposures with sorbed pollutants or microbial biofilms, and environmentally realistic particle sizes and concentrations, and polymer compositions will be essential for relevance to human aging biology [156,204,216].

### 7.3. Longitudinal, One Health, and Exposomics Approaches

Human evidence remains limited to small or cross-sectional biomonitoring studies and lacks long-term epidemiological designs capable of assessing frailty, multimorbidity, cognitive decline, or mortality outcomes across decades [139,143,212,214]. Older adults undergoing rehabilitation often present with reduced physiological reserve and multiple comorbidities, underscoring the need for MNP research to explicitly consider aging populations whose compromised resilience may amplify the functional consequences of chronic environmental exposures. There are few integrative One Health studies tracing particles from environmental reservoirs into food webs and ultimately to human internal dose and biological effect [25,217]. Brain accumulation and potential links to neurodegenerative processes and dementia have emerged as an important research frontier following the recent detection of MPs in human brain tissue, but mechanistic and epidemiological understanding remains limited [139,218]. Integration of exposomics, multi-omics biomarkers of aging, exposure–response modelling, and advanced analytics represents a widely recognized path forward to move beyond association toward causal inference [25,156,198,217].

### 7.4. Future Priorities

Critical next steps include: (1) development and harmonization of high-resolution NP detection with rigorous quality assurance and control; (2) implementation of aging-relevant, disease-sensitive in vitro and in vivo models at environmentally realistic doses; and (3) integration of exposomics and multi-omics platforms to elucidate mechanistic pathways linking MNP exposure to aging and chronic disease [139,156,198,204,217,219]. Additionally, future studies should also incorporate detailed kinetic analyses of adsorption–desorption processes of other pollutants, such as heavy metals, carried by MNPs and intracellular pollutant release, including time-resolved investigations of how particle aging or photo-oxidation alters cargo dynamics and toxicity under realistic exposure conditions [3,43,218,220].

## 8. Conclusions

MNPs are persistent components of the exposome, resulting in continuous exposure across the lifespan. Experimental evidence indicates that these particles engage biological pathways central to aging, including oxidative stress, mitochondrial dysfunction, chronic inflammation, and altered intercellular communication [91]. Aging may increase vulnerability through declining barrier integrity, immune surveillance, and clearance capacity, positioning MNPs as potential modifiers of biological aging within a broader exposomic context [55,221]. Addressing their potential impact requires a One Health approach integrating environmental prevention, ecosystem protection, and aging-focused exposomics research to support healthy aging.

## Figures and Tables

**Table 1:** Micro- and nanoplastic distribution across environmental compartments.

Study/Location	Compartments Sampled	Sampling Design & Methods	MP Concentration (as Reported)	Dominant Size, Shape, Polymer	Key Spatial/Temporal Patterns	Reference
Coastal Istanbul, Marmara Sea (Turkey)	Marine surface sediments (43 stations, 5–70 m depth)	4 seasons; grab sediments; density separation; polymer ID by spectroscopy	Mean (±SD) by season: fall 2000 ± 4100, winter 1600 ± 3900, spring 4300 ± 12,000, summer 9500 ± 20,300 particles/kg DW	Fibres dominant in fall–spring; fragments dominant in summer; 11 polymers, mainly PE (44%) and PP (31%)	Hotspots at river mouths (Golden Horn) and sea-outfall discharges; strong seasonal variation linked to mucilage in summer.	[109]
Yangtze Delta, China (Shanghai & East China Sea)	Urban creeks, rivers, estuary, coastal waters (surface water)	Multi-reach comparison along river–estuary–coast gradient; grab/net surface samples	1.8–2.4 items/L in freshwater creeks/rivers vs. ~0.9 items/L in estuary/coastal waters	Fibers highest in creeks (88%) decreasing seaward; polyesters dominate inland; fibre %: creeks 88%, rivers 81–85%, estuary 66%, coastal 37%	MP abundance decreases from small urban creeks to estuary/coast; downstream increase within rivers toward city centre/estuary.	[110]
Panji coal-mining subsidence area, Anhui (China)	Soil, surface water, sediment	Field survey across mining landscape; density separation, microscopy; polymer ID	Mean abundance: soil 1860.8 n/kg, surface water 11,323.7 n/m^3^, sediment 384.0 n/kg	Predominantly transparent fibres < 0.1 mm; PE common in soil; PP dominant in water and sediment	High MP contamination in all three compartments; distributions correlated with pH, TP, TOC in soil/sediment and with pH, TN, NO_3_-N in water.	[111]
Ebro River Delta, NW Mediterranean (Spain)	River surface water, estuarine benthic sediments, sandy beach sediments	Single-campaign field survey; nets for surface water; grabs/cores for sediments; µ-Raman on subset	Mean abundance: river surface water 3.5 ± 1.4 MPs/m^3^; estuarine benthic sediments 2052 ± 746 MPs/kg DW; sandy beaches 422 ± 119 MPs/kg DW	Fibers ~70 ± 22% of MPs; most particles < 1000 µm, especially 200–500 µm	Estuarine benthic sediments identified as major MP sink; higher MP levels in estuary than adjacent beaches and overlying water.	[112]
Portland, Oregon (USA)	Urban–rural rivers/streams (surface water)	Two watersheds; 3 sampling months (Aug, Sep, Feb); spatial gradient; size-fractionated analysis	Concentrations higher in August (low flow) than February; small (<100 µm) particles more common in August	Fragments most common; gray color dominant; PE most frequent polymer	Negative correlation between MP concentration and flow; positive relation with antecedent 24 h precipitation in wet season; near-stream land use stronger predictor than watershed-scale variables.	[113]
Thulamela Municipality, South Africa	Atmospheric bulk deposition (urban, rural, forest)	Bulk collectors at 3 environments; MPs/m^2^/day; polymer ID	90.51 ± 15.19 to 355.64 ± 47.65 particles/m^2^/day; mean 211.87 ± 31.44 particles/m^2^/day	Mainly transparent fibres; PET dominant	Highest fluxes in urban, lowest in forest; in forest, deposition positively correlated with rainfall, indicating scavenging from air.	[114]
Northern Germany (Hamburg & Mecklenburg-Western Pomerania)	Atmospheric bulk deposition (urban & rural)	11 sites; monthly sampling for 1 year (306 samples); Nile Red staining; µ-Raman on subset; >10 µm	Mean 89 ± 61 MP/m^2^/day	Fibers and fragments; polymer mix characterized by µ-Raman	Significantly higher deposition in urban than rural sites; population density positively related to flux and fibre share; forest canopy “comb-out” effect modifies local deposition.	[115]
Pan-regional, Flanders (Belgium)	Freshwater surface water and sediments	9 locations; 43 water and 9 sediment samples; some resampled under different weather; µFTIR (25–1000 µm)	Surface water: 0–4.8 MP/L (mean 0.48 MP/L); sediments: 0–9558 MP/kg DW (mean 2774.6 ± 2317.9 MP/kg DW)	PS and PP most common polymers; size 25–1000 µm	Large spatial variability; no clear correlation with rainfall, flow, pH, DO, conductivity, or land use; some sediment concentrations above ecological risk thresholds.	[116]
Gallatin River watershed, Montana (USA)	River surface water (mixed land-use catchment)	72 sites; 4 seasons/year over 2 years (714 samples); ~1-L grabs; citizen-science sampling	MPs in 57% samples; mean 1.2 particles/L; majority <-L count range; high temporal variance	Fibers 80%, 0.1–1.5 mm; 93% of analyzed particles synthetic/semi-synthetic	Strong temporal variation; no clear longitudinal or land-use gradient; MP concentration negatively related to discharge at gauged sites (dilution effect).	[117]
Wuhan, Hubei (China)	Urban lakes and river reaches (surface water)	20 lakes plus urban Hanjiang & Yangtze reaches; spatial survey; nets or grabs	1660.0 ± 639.1 to 8925 ± 1591 n/m^3^; highest in Bei Lake	Fibers and coloured plastics; >80% <2 mm; PET & PP dominant	MP abundance in lakes decreases with distance from city centre (*p* < 0.001); rivers generally lower than urban lakes.	[118]
Farmland soils across China (30 sites)	Agricultural soils (vertical profiles)	National-scale survey; 30 farmlands; depth-resolved cores; density separation; spectroscopy	25.56–2067.78 items/kg (mean 358.37 items/kg ≈ 6.79 mg/kg or 0.0007% by mass); MPs ≈93.1% of particles	PP, PE, polyester most common; abundance decreases with depth	Higher soil MP levels in arid/semi-arid north vs. milder southwest; national meta-analysis shows some measured concentrations approach effect thresholds for soil properties and crops.	[119]
Lake Ziway (Ethiopia)	Shoreline sediment; fish (4 species)	Sediment cores + GI tracts of 4 fish species; ATR-FTIR; wet/dry season comparison	Sediment median 30,000 (400–124,000) particles/m^2^ and 764 (0.05–36,233) mg/kg DW; 35% of fish with plastics; median 4 particles/fish (1–26)	PP, PE, alkyd varnish; sizes from micro to small macro; similar log-linear size distributions in fish and sediment	Higher fish ingestion near plastic sources and in wet season; benthic and benthopelagic fish carry more plastics than planktivores; sediment levels exceed some effect thresholds.	[120]
Lake Victoria (Tanzania, Uganda, and Kenya)	Lake surface water (near beaches, rural fish landings, river inflows)	Manta trawl (0.3–4.9 mm) along lake-surface transects; sites grouped by human use	2834–329,167 particles/km^2^ (0.02–2.19 particles/m^2^); highest in urban/recreational beach sites (0.69–2.19 particles/m^2^)	All secondary MPs; 36% < 1 mm; PE and PP dominant	Strong gradient: urban/recreational > rural landings > river-inflow sites; clear influence of local human activity and waste management.	[121]

MP, microplastic; PE, polyethylene; PP, polypropylene; PS, polystyrene; PET, polyethylene terephthalate; DW, dry weight; SD, standard deviation; µm, micrometre; m, metre; kg, kilogram; L, litre; m^3^, cubic metre; MPs/m^2^/day, microplastics per square metre per day; TOC, total organic carbon; TP, total phosphorus; TN, total nitrogen; NO_3_-N, nitrate nitrogen; DO, dissolved oxygen; µFTIR, micro Fourier-transform infrared spectroscopy; ATR-FTIR, attenuated total reflectance Fourier-transform infrared spectroscopy; µ-Raman, micro-Raman spectroscopy.

## Data Availability

No new data were created or analyzed in this study.
